# Supplementary material for: Observation of nuclear-spin Seebeck effect
Source: Nat Commun. 2021 Jul 16;12:4356. doi: 10.1038/s41467-021-24623-6 (PMC8285541; doi:10.1038/s41467-021-24623-6)
Supplement: Supplementary file 1 — Supplementary Information [file 41467_2021_24623_MOESM1_ESM.pdf]

Supplementary Information for

Observation of nuclear-spin Seebeck effect

T. Kikkawa[1,2,3,*], D. Reitz[4], H. Ito[1], T. Makiuchi[1], T. Sugimoto[1], K. Tsunekawa[1], S. Daimon[1], K. Oyanagi[3,5], R. Ramos[2,‡], S. Takahashi[2], Y. Shiomi[6], Y. Tserkovnyak[4], and E. Saitoh[1,2,3,7,8]

1. Department of Applied Physics, The University of Tokyo, Tokyo 113-8656, Japan.

2. WPI Advanced Institute for Materials Research, Tohoku University, Sendai 980-8577, Japan.

3. Institute for Materials Research, Tohoku University, Sendai 980-8577, Japan.

4. Department of Physics and Astronomy, University of California, Los Angeles, California 90095, USA.

5. Faculty of Science and Engineering, Iwate University, Morioka 020-8551, Japan.

6. Department of Basic Science, The University of Tokyo, Tokyo 153-8902, Japan.

7. Advanced Science Research Center, Japan Atomic Energy Agency, Tokai 319-1195, Japan.

8. Institute for AI and Beyond, The University of Tokyo, Tokyo 113-8656, Japan.

[‡] Present address: *Centro de Investigación en Química Biolóxica e Materiais Moleculares (CIQUS), Departamento de Química-Física, Universidade de Santiago de Compostela, Santiago de Compostela 15782, Spain.*

[*]Correspondence and requests for materials should be addressed to T.K. (email: t.kikkawa@ap.t.u-tokyo.ac.jp).

Table of Contents:

Supplementary Note 1 | Magnetization measurement

Supplementary Note 2 | Device characterization

Supplementary Note 3 | Signal characteristics

Supplementary Note 4 | Separation between nuclear-spin Seebeck effect and uni-directional spin Hall magnetoresistance

Supplementary Note 5 | Comments on possible anomalous Nernst effect induced by magnetic proximity effect due to static hyperfine interaction

Supplementary Note 6 | Comparison of voltage normalized by Pt resistance, heating power, and geometric factor between Pt/$MnCO_3$ Devices 1 and 2

Supplementary Note 7 | Comparison of voltage normalized by Pt resistance, heating power, and geometric factor between Pt/$MnCO_3$ Device 2 and Pt/$Y_3Fe_5O_{12}$ (YIG) device

Supplementary Note 8 | Evaluation of temperature difference and nuclear-spin Seebeck thermopower of Pt/$MnCO_3$

Supplementary Note 9 | Theoretical model of nuclear-spin Seebeck effect and comparison with experiment

Supplementary References

Supplementary Note 1 | Magnetization measurement

The magnetization M of the $MnCO_3$ slab was measured using the Vibrating Sample Magnetometer (VSM) option of a Physical Properties Measurement System (PPMS, Quantum Design) in the temperature range from 2 K to 300 K. An external magnetic field, **B**, was applied parallel to the (111) plane of the $MnCO_3$. The M-T curve of the $MnCO_3$ at $B = 25$ mT for $T <$ 60 K is shown in Supplementary Fig. 1, from which the antiferromagnetic ordering (Néel) temperature T_N was estimated to be ~ 35 K.

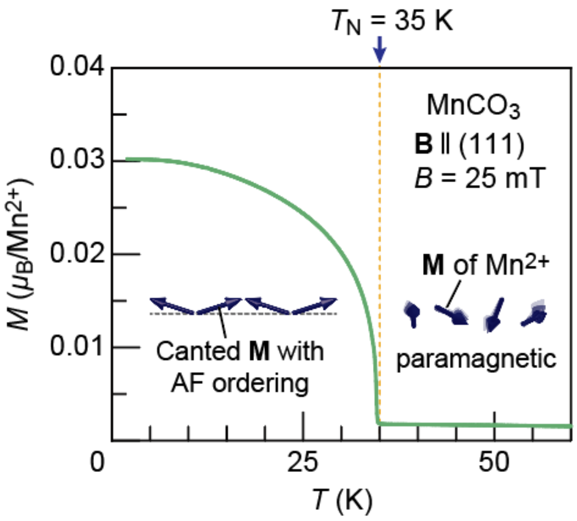

Supplementary Fig. 1 | Magnetic property of $MnCO_3$. Temperature (T) dependence of the magnetization M (M-T curve) of the $MnCO_3$ slab, measured by applying the external magnetic field B = 25 mT parallel to the (111) plane of $MnCO_3$. With decreasing T, the M abruptly increases at around T_N = 35 K. This is due to the antiferromagnetic (AF) ordering of Mn^{2+} magnetization **M**, which are canted slightly from the collinear antiparallel alignment because of the bulk Dzyaloshinskii–Moriya interaction, causing a small net magnetization (~ 0.03 μ_B/Mn^{2+}) (ref.[1]). The canting angle θ is around 0.26° at zero field, which can be modulated by changing B. At B = 14 T, θ ~ 12°.

Supplementary Note 2 | Device characterization

A laser microscope image of a Pt/$MnCO_3$ device, an atomic force microscope image of the (111) surface of the $MnCO_3$, and an X-ray diffraction result of the $MnCO_3$ (111) basal plane are shown in Supplementary Figs. 2a, 2b, and 2c, respectively.

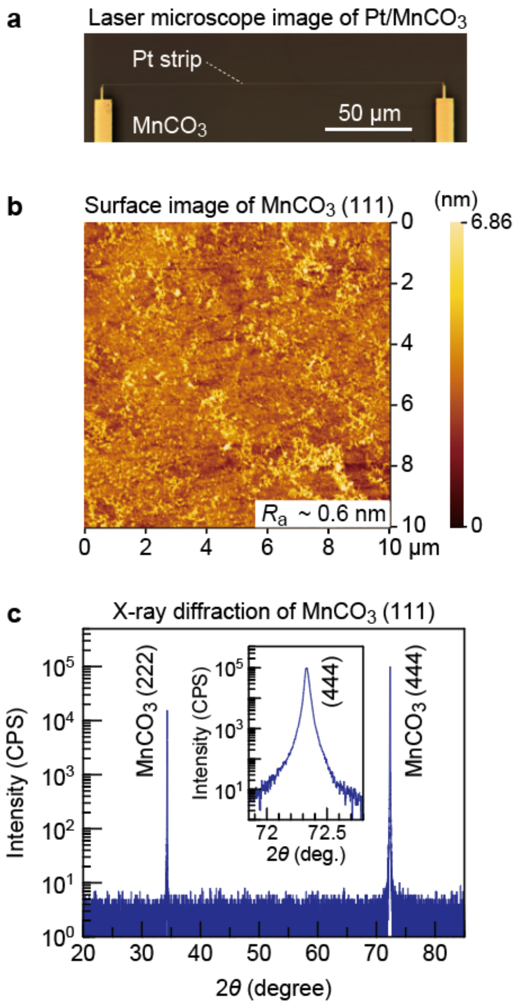

Supplementary Fig. 2 | Microscope image of Pt/$MnCO_3$ device and surface and crystallinity analyses. a, A laser microscope image of a Pt/$MnCO_3$ device. A 10-nm-thick Pt strip (200 μm long and 100 nm wide) is patterned by the electron beam lithography. The white scale bar shows 50 μm. **b**, An atomic force microscope image of the polished (111) surface of $MnCO_3$. The averaged roughness R_a is ~ 0.6 nm, showing nice flatness. **c**, A 2θ-ω X-ray diffraction pattern for a $MnCO_3$ (111) basal plane. CPS in the vertical axis denotes the count per second. Two clear peaks are observed at around 34.2° and 72.3°, which are assigned as the diffraction from the (222) and (444) planes of $MnCO_3$, respectively (in the rhombohedral setting). The inset shows a blowup of the (444) diffraction peak.

Supplementary Note 3 | Signal characteristics

In Supplementary Figs. 3a and 3b, we show the voltage V signal as a function of the applied heat current ($\propto I_{\mathrm{rms}}^2$) in the Pt/$MnCO_3$ Device 1. V appears only when a heat current is applied and the V intensity scales linearly with the heat power $\propto I_{\mathrm{rms}}^2$. As shown in Supplementary Fig. 3c, when **B** is rotated in the xz-plane at an angle α to the x direction, the V signal varies with α in a $\sin\alpha$ pattern and vanishes when $\alpha = 0°$ and 180° ($\mathbf{B} \parallel \pm\mathbf{x}$), consistent with the signal characteristic of the inverse spin-Hall effect (ISHE) induced by the spin Seebeck effect (SSE). We also found that the sign of V reverses when the Pt strip ($\theta_{\mathrm{SHE}} > 0$) is replaced with tungsten exhibiting a negative spin Hall angle θ_{SHE} (refs.[2-4]; see Supplementary Fig. 4).

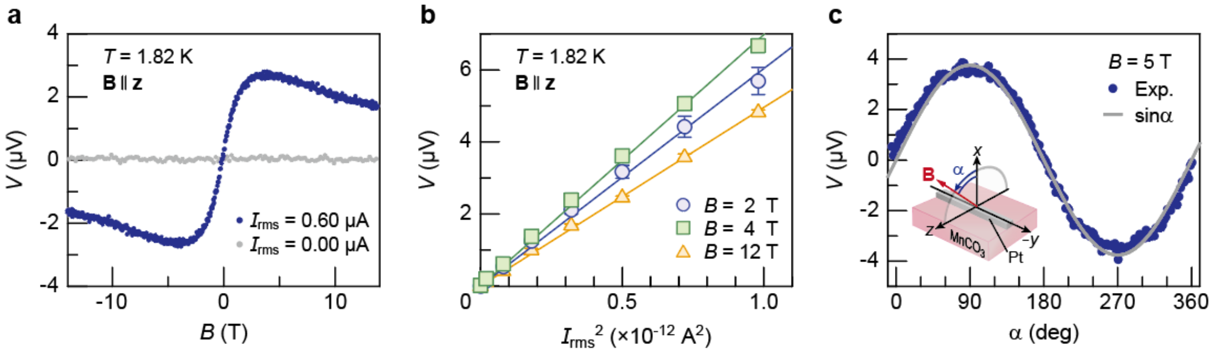

Supplementary Fig. 3 | Voltage characteristics. **a**, B dependence of V for the Pt/$MnCO_3$ Device 1 at T = 1.82 K for the applied current intensity of I_{rms} = 0.60 μA (blue plots) and 0 (gray plots), where the magnetic field **B** is applied along the **z** direction (α = 90°). The V signal disappears in the absence of the applied current. **b**, I_{rms}^2 dependence of V for the same device at T = 1.82 K for several B values (**B** ∥ **z**). The error bar represents the standard deviation. **c**, The magnetic-field angle α dependence of V for the same device at T = 1.82 K and I_{rms} = 0.71 μA (blue plots), where the external field of B = 5 T is rotated in the x-z plane. α denotes the angle between the x axis and the **B** direction. The gray solid curve is a $\sin\alpha$ fit. For the α dependence measurement, the Pt/$MnCO_3$ sample is rotated under a fixed magnetic field B with use of a horizontal rotator option of PPMS.

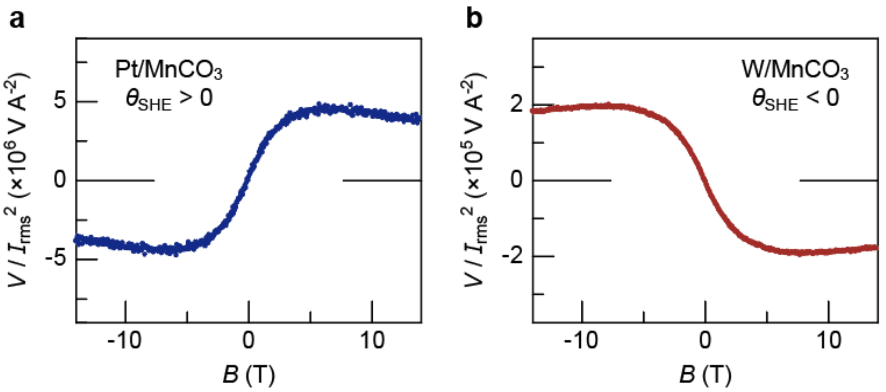

Supplementary Fig. 4 | Comparison of the voltage signal between Pt/$MnCO_3$ and W/$MnCO_3$. **a**,**b**, B dependence of V/I_{rms}^2 at T = 4 K for the Pt/$MnCO_3$ Device 1 (**a**, blue plots) and for the W/$MnCO_3$ device (**b**, red plots). The observed V signal changes its sign when Pt is replaced with W. $\theta_{SHE} > 0$ for Pt and $\theta_{SHE} < 0$ for W (refs.[2-4]).

Supplementary Note 4 | Separation between nuclear-spin Seebeck effect and uni-directional spin Hall magnetoresistance

In our experimental setup for nuclear SSE measurements, an a.c. charge current is applied directly to the Pt wire and the resultant second harmonic voltage V is detected as a function of B. This setup is almost identical to that for the unidirectional spin Hall magnetoresistance (USMR) caused by a current-induced spin accumulation[5-11], whose voltage signal also scales with the applied current squared I_{rms}^2 and shows a B-asymmetric dependence as with the SSE. This means that the separation between the nuclear SSE and USMR is of importance. To separate them, we have performed control experiments and found that the nuclear SSE indeed dominates the observed signal, as elaborated below.

To this end, we first conducted SSE measurements using a chip-heater/Pt/$MnCO_3$ structure, where a resistive chip heater is attached on the Pt layer. In this setup, by applying a charge current to the chip heater (not the Pt layer), a temperature gradient can be created across the Pt/$MnCO_3$ interface, allowing examination of the nuclear SSE free from the possible USMR contribution. Supplementary Fig. 5a shows the B dependence of the second harmonic voltage V generated in the Pt layer at $T = 2$ K measured with a lock-in technique. Voltage signals show up clearly, whose sign changes with respect to the B reversal. Besides, as shown in Supplementary Fig. 5b, the V intensity scales with the applied heating power $R_{\mathrm{h}} I_{\mathrm{rms}}^2$, where R_{h} and I_{rms} are the resistance of the heater ($R_{\mathrm{h}} = 100\ \Omega$) and the amplitude of the applied a.c. charge current ($I_{\mathrm{c}}=\sqrt{2}I_{\mathrm{rms}}\sin\omega t$), respectively. These features are consistent with the characteristics of the ISHE voltage induced by SSE. Furthermore, we found that the temperature T dependence of the signal in this experimental configuration also agrees with that measured for the Pt/$MnCO_3$ devices shown in Fig. 2b in the main text; the SSE signal appears below ~ 10 K and its intensity monotonically increases by decreasing T (see Supplementary Figs. 5c and 5d). The results show that the nuclear SSE manifests in the absence of the external charge current to the Pt layer, which is free from the possible USMR signal.

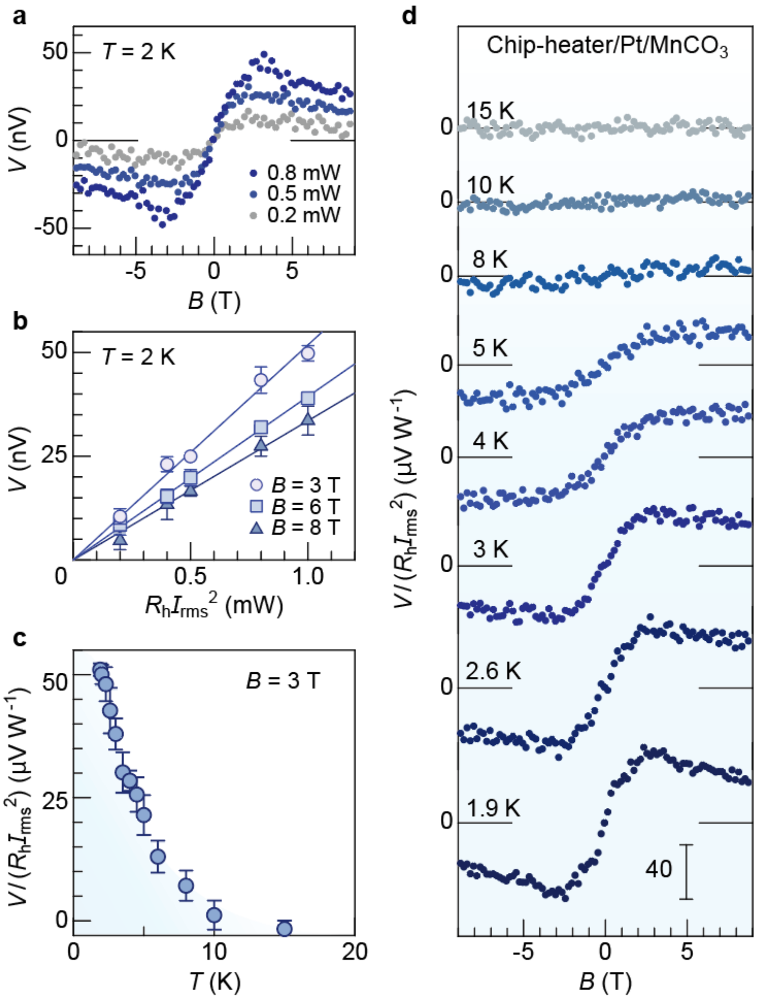

Supplementary Fig. 5 | Nuclear-spin Seebeck effect in chip-heater/Pt/$MnCO_3$ structure. a, *B* dependence of *V* for the chip-heater/Pt/$MnCO_3$ sample at T = 2 K for several heating power $R_h I_{rms}^2$, where R_h and I_{rms} are the resistance of the heater (R_h=100 Ω) and the amplitude of the a.c. charge current, respectively. The sample consists of a 5-nm-thick Pt film sputtered on the whole surface (3×3 mm^2) of a $MnCO_3$ (111) slab with a size of 3×3×0.5 mm^3. Here, on the top of the Pt layer, a resistive heater is attached to create a temperature gradient across the Pt/$MnCO_3$ interface. **b**, Heating power $R_h I_{rms}^2$ dependence of *V* for several *B* values, showing a linear relationship. The error bar represents the standard deviation. **c**, *T* dependence of $V/(R_h I_{rms}^2)$ (voltage *V* normalized by the heating power $R_h I_{rms}^2$) at B = 3 T for 1.9 K < T < 15 K. The error bar represents the standard deviation. **d**, *B* dependence of $V/(R_h I_{rms}^2)$ at selected temperatures.

We then performed another control experiment to evaluate the possible USMR contribution under the experimental setup, where an a.c. charge current (I_c) is applied directly to the Pt layer.

To this end, we followed the experimental method introduced by Avci et al.[5,6] and measured the longitudinal (V_L) and transverse (V_T) voltages of a Hall-bar-shaped Pt/$MnCO_3$ bilayer sample, where V_L and V_T are the second harmonic voltages along the Hall-bar's length ($L = 60$ μm || **y**) and width ($W = 10$ μm || **z**) directions, respectively. In the configuration, if present, the USMR may appear along the Hall-bar's length direction when the in-plane magnetic field B is applied perpendicular to the charge current I_c (**B** || **z**), while it disappears along the Hall-bar's width direction for **B** || **y**. On the other hand, in both the configuration, the SSE-induced second harmonic voltages can show up and their signal-intensity ratio V_L/V_T scales with the geometric factor L/W (refs.[5,6]). By experiments, we indeed observed B-asymmetric second harmonic voltage signals for both the longitudinal V_L for **B** || **z** and transverse V_T for **B** || **y**, whose intensity is proportional to I_{rms}^2. The experimental V_L/V_T ratio was found to be ~ 6.45, which agrees with the geometric factor L/W within ~ 10% accuracy. Therefore, we conclude that most of the second harmonic signal is of SSE origin and not related to the USMR. This conclusion is consistent with the previous similar experiments for a magnetic-insulator $Y_3Fe_5O_{12}$ (YIG) and Pt bilayer reported by Avci et al.[6].

Supplementary Note 5 | Comments on possible anomalous Nernst effect induced by magnetic proximity effect due to static hyperfine interaction

We argue that the anomalous Nernst effect (ANE)[12,13] induced by a magnetic proximity effect (due to static hyperfine interaction) does not explain the observed voltage characteristics. First of all, we observed the non-monotonic B dependence of the voltage, whose intensity increases by increasing B from 0, saturates at around 2 T, and then starts to decrease for further high fields (Fig. 3 in the main text). This non-monotonic behavior is not expected from the ANE scenario as it should monotonically increase with B (ref.[14]) due to the increased nuclear spin polarization along B. On the other hand, the observed B response can be well reproduced by our Korringa spin-current scenario combined with the thermalization model (Fig. 3). Besides, the polarity of V changes in response to the sign of the spin Hall angle θ_{SHE} of metallic layer (Supplementary Fig. 4), consistent with the spin-current scenario, where non-equilibrium spin polarization is created via transverse components of interfacial hyperfine interaction. We thus conclude that the nuclear SSE governs the observed signal, rather than the possible proximity ANE.

Supplementary Note 6 | Comparison of voltage normalized by Pt resistance, heating power, and geometric factor between Pt/$MnCO_3$ Devices 1 and 2

In the main text, we show the voltage V normalized by the applied charge current squared I_{rms}^2. As shown in Fig. 2, the obtained V/I_{rms}^2 value for the Pt/$MnCO_3$ Device 1 is one order of magnitude higher than that for Device 2. We attribute the difference mainly to that of the electrical resistance R_{Pt} of the Pt layer; the R_{Pt} value for Device 1 at $T = 1.8$ K is ~ 179 kΩ, several times higher than that for Device 2 at the same temperature (R_{Pt} ~ 30.9 kΩ). The output SSE signal indeed scales with $R_{\mathrm{Pt}}^2 I_{\mathrm{rms}}^2 l^{-1}$, since the electromotive force induced by the ISHE is proportional to R_{Pt} (ref.[15]) and the input heating power in our experimental setup increases proportionally to $R_{\mathrm{Pt}} I_{\mathrm{rms}}^2$ (l is the Pt length along the electrode direction and is the same between Devices 1 and 2) [for details see Eq. (3) in Supplementary Note 9]. As shown in Supplementary Fig. 6, the $Vl/(R_{\mathrm{Pt}}^2 I_{\mathrm{rms}}^2)$ intensity is almost identical between the Pt/$MnCO_3$ Devices 1 and 2 [$V_{\mathrm{max}} l/(R_{\mathrm{Pt}}^2 I_{\mathrm{rms}}^2)$ ~ 48 nAmW^{-1} for Device 1 at $T = 1.82$ K and ~ 38 nAmW^{-1} for Device 2 at $T = 1.80$ K]. This shows that the quantity $Vl/(R_{\mathrm{Pt}}^2 I_{\mathrm{rms}}^2)$ is a nice benchmark to compare the SSE performance with different samples, in which the information of the device resistance and geometry is taken into account. We would like to note that the magnetoresisitance (MR) ratio of our Pt film is as small as ~ 0.02% up to the B intensity of 14 T at $T = 2$ K (Supplementary Fig. 7), meaning that the heating power $R_{\mathrm{Pt}} I_{\mathrm{rms}}^2$ does not depend on B for the whole B range in the present study.

The difference of R_{Pt} may originate from that of the Pt width; although it was designed to be 100 nm, the resultant Pt width was found to be ~ 30 nm and ~ 97 nm for Devices 1 and 2, respectively, from atomic force microscopy measurements. This may be due to the different type of electron-beam resist PMMA (polymethyl methacrylate) used for making Devices 1 and 2; Device 1 was made with 950 PMMA "A4" resist, while Device 2 with 950 PMMA "A2" (KAYAKU Advanced Materials, Inc.). According to the data sheet from the provider (ref.[16]), due to their different anisole content, the A4-type resist layer becomes about three times thicker than the A2-type after a spin-coating process. This may cause possible underdose in the electron-beam lithography process for Device 1, resulting in the narrow width for the Pt pattern

of Device 1. Nevertheless, we would like to note that the Pt resistance difference can be taken into account in our analysis and does not affect our conclusion.

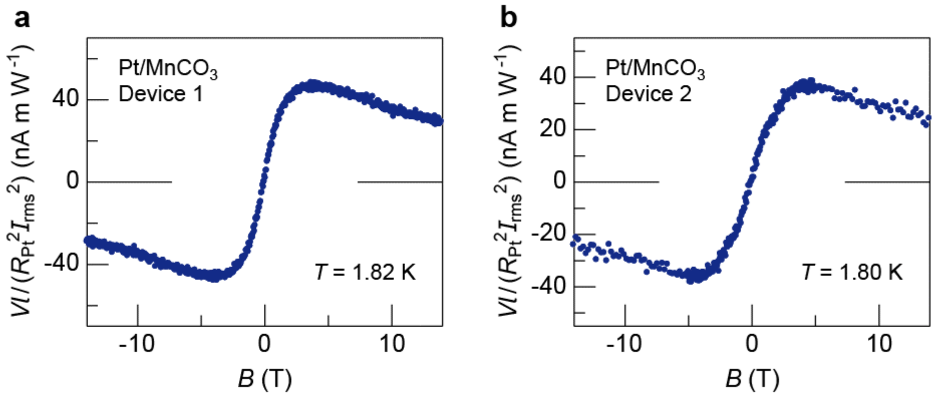

Supplementary Fig. 6 | Comparison of signal intensity between Pt/$MnCO_3$ Devices 1 and 2. a,**b**, *B* dependence of $Vl/(R_{Pt}^2 I_{rms}^2)$ (voltage *V* normalized by the Pt resistance R_{Pt}, heating power $R_{Pt}I_{rms}^2$ applied to the Pt wire, and the inverse of the Pt length along the electrode direction l^{-1}) for the (**a**) Pt/$MnCO_3$ Device 1 at *T* = 1.82 K and (**b**) Device 2 at *T* = 1.80 K.

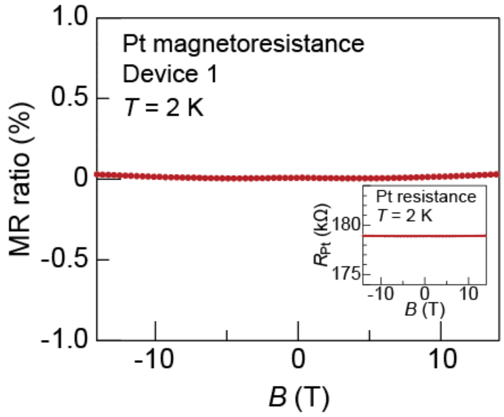

Supplementary Fig. 7 | Magnetoresistance of Pt wire. *B* dependence of the magnetoresistance (MR) ratio relative to zero field for the Pt wire of Device 1 at *T* = 2 K, where the in-plane *B* is applied perpendicular to the Pt wire. The inset shows the *B* dependence of electric resistance R_{Pt} of the Pt wire at the same temperature.

Supplementary Note 7 | Comparison of voltage normalized by Pt resistance, heating power, and geometric factor between Pt/$MnCO_3$ Device 2 and Pt/$Y_3Fe_5O_{12}$ (YIG) device

We compare the $Vl/(R_{Pt}^2 I_{rms}^2)$ value between the Pt/$MnCO_3$ Devices 2 at T = 101 mK and a Pt/YIG-film device at 300 K having the same electrode and heater dimensions. Here, the single-crystalline YIG-film with the thickness of ~ 4 μm is grown on a $Gd_3Ga_5O_{12}$ (111) substrate with liquid phase epitaxy. The maximum output $V_{max}l/(R_{Pt}^2 I_{rms}^2)$ value is ~ 58 nAmW^{-1} for the Pt/$MnCO_3$ Device 2 as shown in Supplementary Fig. 8a, which is nearly two orders of magnitude higher than that for the Pt/YIG (~ 1 nAmW^{-1}; Supplementary Fig. 8b).

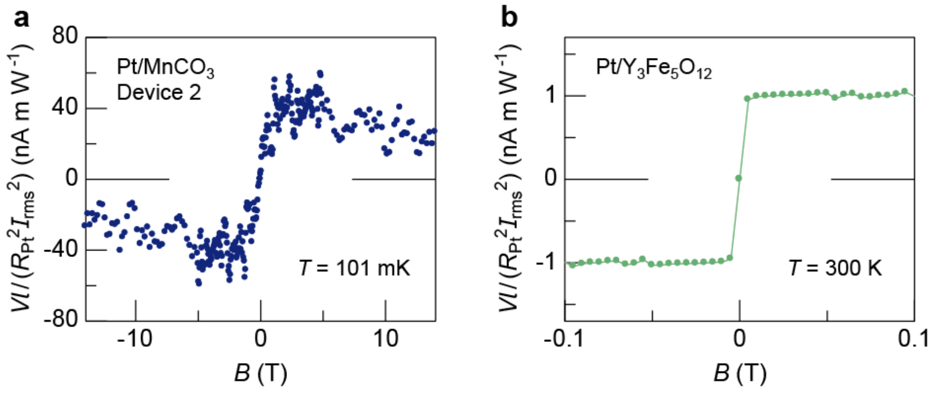

Supplementary Fig. 8 | Comparison of signal intensity between Pt/$MnCO_3$ Device 2 and Pt/$Y_3Fe_5O_{12}$. a, B dependence of $Vl/(R_{Pt}^2 I_{rms}^2)$ for the Pt/$MnCO_3$ Device 2 at T = 101 mK. **b**, B dependence of $Vl/(R_{Pt}^2 I_{rms}^2)$ for the Pt/YIG-film at T = 300 K. Here, the YIG-film with the thickness of ~ 4 μm is grown on a $Gd_3Ga_5O_{12}$ substrate with liquid phase epitaxy. The Pt layer for the Pt/$MnCO_3$ Device 2 and Pt/YIG has the same dimensions.

Supplementary Note 8 | Evaluation of temperature difference and nuclear-spin Seebeck thermopower of Pt/$MnCO_3$

For the nuclear SSE measurement on the Pt/$MnCO_3$ Device 2 at the lowest temperature of T = 101 mK, the intensity of applied a.c. charge current I_{rms} is as small as 70 nA, which gives the heating power of $R_{Pt}I_{rms}^2$ = 154 pW. This value is several orders of magnitude smaller than the cooling power of our dilution fridge: 200 μW at 100 mK. Indeed, with the application of such small heating power during the measurement, there was no change of the sample's temperature monitored by a RuO_x sensor, which was attached to the back of the oxygen-free high thermal conductivity (OFHC grade) copper plate, on which the Pt/$MnCO_3$ sample was mounted. We evaluated the temperature difference ΔT generated in the sample to be of the order of ~ 0.3 mK by a heat-flow analysis[17], where the Pt wire is modelled as a heat source embedded in the center of a half cylinder of $MnCO_3$ (with a radius of 0.5 mm) exhibiting a thermal conductivity value of 8.8×10^{-6} $Wcm^{-1}K^{-1}$ at 100 mK which is estimated from the experiment by Ozhogin et al.[18].

Using the above estimated temperature difference $\Delta T \sim 0.3$ mK, we extract the spin-Seebeck thermopower S defined as $S = (V/L_V)/(\Delta T/L_T)$ (ref.[19]), which is most frequently used in the SSE research community. Here, L_V and L_T represent the length along the electrode direction and the thickness of a SSE device, respectively. For the Pt/$MnCO_3$ Device 2 at T = 101 mK, S is evaluated as ~ 11 μV K^{-1}, which is nearly two orders of magnitude higher than that for 10-nm-thick Pt/YIG-slab junctions measured at room temperature ($S \sim 0.2 - 0.5$ μV K^{-1}) (refs.[12,13]), a situation consistent with the comparison shown in Supplementary Fig. 8.

Supplementary Note 9 | Theoretical model of nuclear-spin Seebeck effect and comparison with experiment

The electronic SSE is driven by both an interfacial temperature difference between magnons and electrons in the metal[20] and a non-equilibrium chemical potential which builds up at the interface due to a bulk thermal gradient[21-24]. While electronic magnons can transport spin due to their finite group velocities and lifetimes, nuclear spin transport is limited to small fields since they have almost zero group velocity aside from the small hybridized region of the Brillouin zone, as can be seen from Fig. 1c in the main text. Furthermore, since here the magnetic field is perpendicular to the Néel order (and the nuclei align locally with the electronic spins), spin is not conserved along the field. Thus, we formulate the nuclear SSE in terms of interfacial thermal spin pumping rather than bulk spin transport.

The measured nuclear SSE voltage, normalized by the input energy flux, reported here appears to be larger than one might expect when compared to, for example, YIG at room temperature in a device with the same geometry. There are several quantities relevant to the scaling of the SSE with temperature: the metal's resistivity, spin-diffusion length, and spin Hall angle; the thermal conductivities, phonon inelastic equilibration and phonon-spin carrier relaxation lengths[25]; and the magnetic dynamics itself via the spin Seebeck coefficient $\mathcal{S}$. At low temperatures the phonons become essentially noninteracting and thus equilibrate very poorly to a common temperature; then for a fixed energy flux, the bulk thermal gradient and temperature drop across the interface increase due to smaller thermal bulk and interfacial conductivities. Additionally, the interfacial temperature drop may be effectively larger due to a longer inelastic phonon equilibration length. Furthermore, since the nuclear spins are all thermalized $\mathcal{S}_\mathrm{n} \propto 1/T$ as compared to the magnonic SSE which freezes out as $\mathcal{S}_\mathrm{m} \propto T^{3/2}$ for ferromagnets (both bulk and interfacial contributions[26]) and $\mathcal{S}_\mathrm{m} \propto T^3$ for this antiferromagnet (see below). Comparing the nuclear SSE contribution to ferromagnetic and antiferromagnetic contributions, at $T \gg T_\mathrm{C}$ or T_N we would expect the paramagnetic contributions to show a similar $1/T$ scaling, at which point it should dominate over the nuclear's since interfacial electron exchange coupling is much stronger than the interfacial nuclear hyperfine coupling (T_C and T_N are the Curie and Néel temperatures, respectively). From $T \sim T_\mathrm{C}$ or T_N and down, however, the magnonic SSE is reduced, while the nuclear one keeps increasing as $1/T$.

Each nuclear spin aligns antiparallel to the electronic sublattice magnetization on the same site, and the net spin current across two adjacent sites cancels unless there is canting of the electronic spins (with angle $\theta = \chi b$, see Fig. 1d in the main text). We calculate the nuclear spin current transferred into the metal due to Korringa-like relaxation[27] (depicted in Fig. 1a) by Fermi's Golden rule in the limit $k_B T \gg \hbar\omega_n$:

$$J_n = \chi b J_{ne} = \rho(\epsilon_F)^2 a^2 \pi \chi b \hbar\omega_n (T_e - T_n)/T \tag{1}$$

where J_{ne} is the spin current per site and J_n is the average over a pair of sites, $\rho(\epsilon_F)$ is the density of states at the Fermi level in units of $(\text{energy}\cdot\text{volume})^{-1}$, a is the interfacial hyperfine interaction constant between nuclei and the spin density in the metal, and T is the average temperature. The temperature dependence in J_{ne} differs from the usual Korringa spin-relaxation rate, $\tau_k^{-1} \propto T$, since J_{ne} is due to the spin flow, minus the backflow, into the Fermi gas. We define the nuclear-electron spin Seebeck coefficient per site Γ_{ne} by $J_{ne} \equiv \Gamma_{ne} k_B (T_e - T_n)$ and define the nuclear spin-mixing conductance per unit area as $g_n^{\uparrow\downarrow} \equiv 4\pi s_n \rho(\epsilon_F)^2 a^2$ for saturated nuclear spin density s_n ($s_n \equiv I/\mathcal{A}$, calculated for spin $I = 1/2$ and interfacial area $\mathcal{A}$ per site), in analogy with the electronic result in ref.[28].

The nuclear spin Seebeck coefficient $\mathcal{S}_n$ relates the experimentally-applied thermal bias $T_e - T_p$ to the spin current density $J_n/\mathcal{A} \equiv \mathcal{S}_n k_B (T_e - T_p)$ and involves balancing, for each nuclear site at the interface, the spin flow from phonons in the antiferromagnet to electrons in the metal. When the temperature is well above the magnon gap ω_{m0}, the magnons can directly transfer the small energy $\hbar\omega_n$ to the nuclei by two-magnon (Raman-like absorption and remission) scattering processes in the magnon continuum[29]; rapidly equilibrating the phonon, magnon, and nuclear temperatures. At temperatures comparable to and below the magnon gap, the mechanism driving the nuclear-phonon spin current $J_{np} \equiv \Gamma_{np} k_B (T_n - T_p)$ involves a virtual magnon process which mediates a nuclear spin flip by transmitting energy $\hbar\omega_n$ from phonons. This occurs near the Gamma point since ω_n is small on the scale of the magnon and phonon dispersions. We diagonalize the magnon-phonon Hamiltonian by performing a Bogoliubov transformation which yields two hybridized branches. The hyperfine interaction in the antiferromagnet now becomes a second-order, direct nuclear coupling to the hybridized

field operators. Then to lowest order in magnon hybridization within the low-energy branch, we get by Fermi's Golden rule: $\Gamma_{\mathrm{np}} \propto 1/T\omega_{\mathrm{m0}}^2$, where $\omega_{\mathrm{m0}} = \gamma_{\mathrm{e}}\sqrt{B_{a'}^2 + B^2}$ is the magnon gap for easy-axis anisotropy field[30] $B_{a'}$ and perpendicular applied field, both within the easy plane. The remaining B,T-independent coefficient C is taken from experiment: $\Gamma_{\mathrm{np}}/\Gamma_{\mathrm{ne}} = C/\omega_{\mathrm{m0}}^2$. We fit C by aligning the experimental and theoretical crossover fields B_{c}, Thus, in our theory $\mathcal{S}_{\mathrm{n}}$ is rate-limited by thermalization between nuclei and electrons when $B < B_{\mathrm{c}}$, and thermalization between nuclei and phonons when $B > B_{\mathrm{c}}$.

The magnonic spin Seebeck coefficient $\mathcal{S}_{\mathrm{m}}$ is defined in the same way as $\mathcal{S}_{\mathrm{n}}$, relating the spin current density $J_{\mathrm{m}} \equiv \mathcal{S}_{\mathrm{m}} k_{\mathrm{B}}(T_{\mathrm{e}} - T_{\mathrm{p}})$ to the thermal bias[31]. We calculate $\mathcal{S}_{\mathrm{m}}$ semi-classically using the fluctuation-dissipation theorem, assuming high magnon quality factors as in ref.[32], to get

$$\mathcal{S}_{\mathrm{m}} = \frac{g_{\mathrm{m}}^{\uparrow\downarrow}\hbar\chi b}{2\pi s_{\mathrm{e}} k_{\mathrm{B}}} \int \frac{d^3k}{(2\pi)^3} \omega_{\mathrm{m}k} \partial_T n_{\mathrm{BE}}(\omega_{\mathrm{m}k}) \quad (2)$$

where $g_{\mathrm{m}}^{\uparrow\downarrow}$ is the magnonic interfacial spin-mixing conductance per unit area, s_{e} is the saturated spin density in the antiferromagnet ($s_{\mathrm{e}} \equiv S/V$, for dimensionless spin S and volume V per site), $n_{\mathrm{BE}}(\omega_{\mathrm{m}k}) = [\exp(\hbar\omega_{\mathrm{m}k}/k_{\mathrm{B}}T) - 1]^{-1}$ is the Bose–Einstein distribution function, and $\omega_{\mathrm{m}k} = \sqrt{\gamma_{\mathrm{e}}{}^2(B_{a'}^2 + B^2) + c^2k^2}$ where c is the speed of antiferromagnetic spin waves at large wave numbers k. $\mathcal{S}_{\mathrm{m}}$ may be evaluated analytically in the limit $k_{\mathrm{B}}T \gg \hbar\omega_{\mathrm{m}k}$ which gives $\mathcal{S}_{\mathrm{m}} \propto BT^3$. At low temperatures and large fields, magnon thermal populations at ω_{m0} are exponentially suppressed[31], causing $\mathcal{S}_{\mathrm{m}}$ to decrease monotonically with increasing field. The transition in field behavior is roughly marked by $\hbar\gamma_{\mathrm{e}}B_{\mathrm{c}} \sim k_{\mathrm{B}}T$. Finally, there is an additional type of contribution to the electronic SSE that is due to the hybridization of nuclei and magnons, which is known as nuclear frequency pulling[33]. However in our system this can be shown to decrease more slowly with increasing field than $\mathcal{S}_{\mathrm{m}}$, but to be negligible compared to $\mathcal{S}_{\mathrm{n}}$.

The voltage measured due to the SSE arises from the inverse spin Hall effect associated with the thermally-induced spin current[34]. When the SSE voltage (normalized by the injector current squared) is driven by an interfacial temperature discontinuity, it is given by[32]

$$\frac{V_{\rm SSE}}{I_{\rm c}^2} = \frac{\mathcal{S}_i(B,T)}{\kappa^*(T)}\frac{2ek_{\rm B}}{\hbar}\frac{\lambda^*}{l}R_{\rm d}R_{\rm h} \tag{3}$$

where $\lambda^* \equiv \lambda_{\rm sd}\theta_{\rm SHE}$ is the effective spin-diffusion length times the detector's spin Hall angle $\theta_{\rm SHE} \equiv \hbar J_{\rm c}/2eJ_{\rm s}$ for lateral charge density $J_{\rm c}$ and interfacial spin current density $J_{\rm s}$, with $J_{\rm s} = J_{\rm n}/\mathcal{A}$ for the nuclei and $J_{\rm s} = J_{\rm m}$ for the magnons, l is the length of the detector, $R_{\rm h}$, $R_{\rm d}$ is the resistance in the metal heater and detector, κ^* is the effective phononic interfacial Kapitza conductance (which relates the injected heat flux to $T_{\rm e} - T_{\rm p}$ at the interface), and $\mathcal{S}_i$ is the local Seebeck coefficient in units of inverse area (i = n, m). In order to fit the overall common factors (including the device resistance) multiplied by $\mathcal{S}_i$ in $V_{\rm SSE}$, we use the low-field slope $f(T)$ where both theory and experiment are linear in B. The slope goes as $f(T) \propto 1/(a + bT^c)$ where $a = 8.4$, $b = 2.2$, and $c = 1.6$. Since at small fields $\mathcal{S}_{\rm m} \propto BT^3$ and $\mathcal{S}_{\rm n} \propto B/T$, for the electronic SSE we would need $\kappa^*_{\rm m}(T) \propto (a + bT^c)T^3$ and for the nuclear SSE we would need $\kappa^*_{\rm n}(T) \propto (a + bT^c)/T$ to fit the experimental $f(T)$. The theoretical curves plotted in Fig. 3 for comparison to the data are calculated as $\mathcal{S}_{\rm n}f(T)/f_{\rm n}(T)$ and $\mathcal{S}_{\rm m}f(T)/f_{\rm m}(T)$, where $f_i(T) \equiv \partial\mathcal{S}_i/\partial B(B = 0)$ are the slopes of the theoretical Seebeck coefficients evaluated at zero field.

While our theory for $\mathcal{S}_{\rm n}$ reproduces most aspects of the measured signal, it slightly underestimates the data at large relative to small fields (Fig. 3e). This might be explained by additional nuclear-phonon thermalization channels such as direct nuclear-phonon coupling or indirect coupling via the second magnon branch associated with Néel excitations out of the easy plane. Since $\mathcal{S}_{\rm n}$ is limited by $\Gamma_{\rm np}$ at $B > B_{\rm c}$, our theory would then give a lower bound on $\mathcal{S}_{\rm n}$ there, which is consistent with experiment, if the additional channels do not decrease as fast with field as our $\Gamma_{\rm np}$. Additional, inelastic channels for nuclear-electron spin transport may also affect the fit of $f_{\rm n}(T)$ to $f(T)$, which comes from the data at $B < B_{\rm c}$ where $\Gamma_{\rm ne}$ limits $\mathcal{S}_{\rm n}$, and the fit parameter C which controls the position of $B_{\rm c}$. In our theory, the contributions to $\Gamma_{\rm np}$ and $\Gamma_{\rm ne}$ have the same overall temperature dependence giving a T-independent $B_{\rm c}$, which is consistent with the data over a large range of temperatures.

Supplementary References

1. Borovik-Romanov, A. S. Investigation of weak ferromagnetism in the $MnCO_3$ single crystal. *J. Exptl. Theoret. Phys.* **36**, 766–781 (1959).

2. Uchida, K. et al. Longitudinal spin Seebeck effect: from fundamentals to applications. *J. Phys.: Condens. Matter* **26**, 343202 (2014).

3. Pai, C.-F. et al. Spin transfer torque devices utilizing the giant spin Hall effect of tungsten. *Appl. Phys. Lett.* **101,** 122404 (2012).

4. Sinova, J., Valenzuela, S. O., Wunderlich, J., Back, C. H. & Jungwirth, T. Spin Hall effects. *Rev. Mod. Phys.* **87**, 1213–1259 (2015).

5. Avci, C. O. et al. Unidirectional spin Hall magnetoresistance in ferromagnet/normal metal bilayers. *Nat. Phys.* **11**, 570–575 (2015).

6. Avci, C. O. et al. Magnetoresistance of heavy and light metal/ferromagnet bilayers. *Appl. Phys. Lett.* **107**, 192405 (2015).

7. Yasuda, K. et al. Large unidirectional magnetoresistance in a magnetic topological insulator. *Phys. Rev. Lett.* **117**, 127202 (2016).

8. Zhang, S. S.-L. & Vignale, G. Theory of unidirectional spin Hall magnetoresistance in heavy-metal/ferromagnetic-metal bilayers. *Phys. Rev. B* **94**, 140411(R) (2016).

9. Avci, C. O. et al. Origins of the unidirectional spin Hall magnetoresistance in metallic bilayers. *Phys. Rev. Lett.* **121**, 087207 (2018).

10. Kim, K.-J. et al. Possible contribution of high-energy magnons to unidirectional magnetoresistance in metallic bilayers. *Appl. Phys. Exp.* **12**, 063001 (2019).

11. Sterk, W. P., Peerlings, D. & Duine, R. A. Magnon contribution to unidirectional spin Hall magnetoresistance in ferromagnetic-insulator/heavy-metal bilayers. *Phys. Rev. B* **99**, 064438 (2019).

12. Kikkawa, T. et al. Longitudinal spin Seebeck effect free from the proximity Nernst effect. *Phys. Rev. Lett.* **110**, 067207 (2013).

13. Kikkawa, T. et al. Separation of longitudinal spin Seebeck effect from anomalous Nernst effect: Determination of origin of transverse thermoelectric voltage in metal/insulator junctions. *Phys. Rev. B.* **88**, 214403 (2013).

14. Ikhlas, M. et al. Large anomalous Nernst effect at room temperature in a chiral antiferromagnet. *Nat. Phys.* **13**, 1085–1090 (2017).

15. Schreier, M. et al. Sign of inverse spin Hall voltages generated by ferromagnetic resonance and temperature gradients in yttrium iron garnet platinum bilayers. *J. Phys. D: Appl. Phys.* **48**, 025001 (2015).
16. https://kayakuam.com/wp-content/uploads/2020/07/KAM-PMMA-TDS.4.12.21.pdf
17. Baehr H. D. & Stephan K. *Heat and Mass Transfer, Third revised edition* (Springer-Verlag, Berlin Heidelberg, 2011).
18. Ozhogin, V. I., Inyushkin, A. V. & Babushkina, N. A. Thermal conductivity of easy-plane antiferromagnets. *J. Magn. Magn. Mater.* **31–34**, 147–148 (1983).
19. Uchida, K. et al. Thermoelectric generation based on spin Seebeck effects. *Proc. IEEE* **104**, 1946–1973 (2016).
20. Xiao, J., Bauer, G. E. W., Uchida, K., Saitoh, E. & Maekawa, S. Theory of magnon-driven spin Seebeck effect. *Phys. Rev. B* **81**, 214418 (2010).
21. Rezende, S. M. et al. Magnon spin-current theory for the longitudinal spin-Seebeck effect. *Phys. Rev. B* **89**, 014416 (2014).
22. Cornelissen, L. J., Peters, K. J. H., Bauer, G. E. W., Duine, R. A. & van Wees, B. J. Magnon spin transport driven by the magnon chemical potential in a magnetic insulator. *Phys. Rev. B* **94**, 014412 (2016).
23. Rezende, S. M., Rodríguez-Suárez, R. L., Cunha, R. O., Lopez Ortiz, J. C., & Azevedo, A. Bulk magnon spin current theory for the longitudinal spin Seebeck effect. *J. Magn. Magn. Mater.* **400**, 171–177 (2016).
24. Rezende, S. M. *Fundamentals of Magnonics* (Springer Nature Switzerland AG, Switzerland, 2020).
25. Prakash, A. et al. Evidence for the role of the magnon energy relaxation length in the spin Seebeck effect. *Phys. Rev. B* **97**, 020408(R) (2018).
26. Flebus, B., Bender, S. A., Tserkovnyak, Y. & Duine, R. A. Two-fluid theory for spin superfluidity in magnetic insulators. *Phys. Rev. Lett.* **116**, 117201 (2016).
27. Korringa, J. Nuclear magnetic relaxation and resonance line shift in metals. *Physica* **16**, 601–610 (1950).
28. Bender, S. A. & Tserkovnyak, Y. Interfacial spin and heat transfer between metals and magnetic insulators. *Phys. Rev. B* **91**, 140402(R) (2015).
29. Flebus, B. & Tserkovnyak, Y. Quantum-impurity relaxometry of magnetization dynamics.

Phys. Rev. Lett. **121**, 187204 (2018).
30. Fink, H. & Shaltiel, D. Nuclear frequency pulling in a Dzialoshinskii-Moriya-type weak ferromagnet: $MnCO_3$. *Phys. Rev.* **136**, A218–A222 (1964).
31. Hoffman, S., Sato, K. & Tserkovnyak, Y. Landau-Lifshitz theory of the longitudinal spin Seebeck effect. *Phys. Rev. B.* **88**, 064408 (2013).
32. Reitz, D., Li, J., Yuan, W., Shi, J. & Tserkovnyak, Y. Spin Seebeck effect near the antiferromagnetic spin-flop transition. *Phys. Rev. B* **102**, 020408 (2020).
33. Shiomi, Y. et al. Spin pumping from nuclear spin waves. *Nat. Phys.* **15**, 22–26 (2019).
34. Uchida, K. et al. Thermal spin pumping and magnon-phonon-mediated spin-Seebeck effect. *J. Appl. Phys.* **111**, 103903 (2012).
